# Ultrasensitive gold micro-structured electrodes enabling the detection of extra-cellular long-lasting potentials in astrocytes populations

**DOI:** 10.1038/s41598-017-14697-y

**Published:** 2017-10-27

**Authors:** Ana L. G. Mestre, Mónica Cerquido, Pedro M. C. Inácio, Sanaz Asgarifar, Ana S. Lourenço, Maria L. S. Cristiano, Paulo Aguiar, Maria C. R. Medeiros, Inês M. Araújo, João Ventura, Henrique L. Gomes

**Affiliations:** 1 Universidade do Algarve, Faculdade de Ciências e Tecnologia, 8005-139 Faro, Portugal; 2 Instituto de Telecomunicações, Avenida Rovisco Pais 1, 1049-001 Lisboa, Portugal; 3 Instituto de Física dos Materiais da Universidade do Porto, Instituto de Nanociências e Nanotecnologia, Departamento de Física e Astronomia, Universidade do Porto, Rua do Campo Alegre 687, 4169-007 Porto, Portugal; 4 Departamento de Ciências Biomédicas e Medicina, Universidade do Algarve, 8005-139 Faro, Portugal; 5 Centro de Investigação em Biomedicina, Universidade do Algarve, 8005-139 Faro, Portugal; 6 Centro de Ciências do Mar, Universidade do Algarve, 8005-139 Faro, Portugal; 7 Instituto de Engenharia Biomédica, Universidade do Porto, Rua Alfredo Allen 208, 4200-135 Porto, Portugal; 8 Instituto de Investigação e Inovação em Saúde, Universidade do Porto, Rua Alfredo Allen 208, 4200-135 Porto, Portugal; 9 Instituto de Telecomunicações, Departamento de Engenharia Electrotécnica e Computadores, Universidade de Coimbra, 3030-290 Coimbra, Portugal

## Abstract

Ultra-sensitive electrodes for extracellular recordings were fabricated and electrically characterized. A signal detection limit defined by a noise level of 0.3–0.4 μV for a bandwidth of 12.5 Hz was achieved. To obtain this high sensitivity, large area (4 mm^2^) electrodes were used. The electrode surface is also micro-structured with an array of gold mushroom-like shapes to further enhance the active area. In comparison with a flat gold surface, the micro-structured surface increases the capacitance of the electrode/electrolyte interface by 54%. The electrode low impedance and low noise enable the detection of weak and low frequency quasi-periodic signals produced by astrocytes populations that thus far had remained inaccessible using conventional extracellular electrodes. Signals with 5 μV in amplitude and lasting for 5–10 s were measured, with a peak-to-peak signal-to-noise ratio of 16. The electrodes and the methodology developed here can be used as an ultrasensitive electrophysiological tool to reveal the synchronization dynamics of ultra-slow ionic signalling between non-electrogenic cells.

## Introduction

Substrate integrated planar microelectrode arrays (MEAs)^1–3^ are considered the standard electrophysiological methodology for long-term analysis of *in vitro* neuronal cells and networks. MEA technology provides spatial resolution, multi-unit electrical recordings and a mean for the electrical stimulation of neuronal cells. Currently available MEA electrodes have areas typically below 1,000 μm^2^ and generate a thermal noise in the range of 5–20 μV. This intrinsic noise defines the system detection limit. In voltage measurements, the electrode thermal noise is inversely proportional to the surface area. Increasing the area of the electrodes causes an increase in sensitivity due to the reduction in noise and impedance. However, as the area increases the ability to discriminate between individual cell signals is impaired. To circumvent this trade-off between electrode sensitivity and spatial resolution several laboratories have started to develop structured electrodes. These emergent electrodes make use of vertical micro or nano-structures^4,5^ coated, namely, with carbon nanotubes^6–8^, or conducting polymers^9–18^, which provide a softer and more roughened surface. The strategy behind these approaches is to increase the effective area and to promote a more intimate interaction of the electrode with cells. A well-known example is the use of micron size gold mushroom-like shapes^19,20^. It has been shown that cells engulf the protruding gold mushroom-like shapes, lowering the impedance and improving the electrical coupling between the cells and the electrode.

Such micro-structured electrodes have been optimized to record action potentials. However, there is some less known electrical activity produced by cells and organs with important physiological/functional roles. This activity generates weak and long lasting electrical perturbations that propagate trough the extracellular milieu^19^. They are caused by ions, polar molecules or zwitterions that can pass from cell to cell by gap junctions. These fluctuations are steady or slow changing gradients and they progress thousands of times more slowly than action potentials. This activity does not show spikes but smooth signals that can change over a period of time, from several seconds to minutes, and often are a result of cell cooperative phenomena. Astrocytes are cells that generate such type of long lasting signals. Patch clamp recordings and optical fluorescence methods have revealed interesting functional electrical properties in astrocytes but these mechanisms are not yet fully understood^21–25^.

Although MEA technology has not been optimized to record long lasting signals produced by astrocytes, there are few studies reporting astrocyte activity using MEAs. In a study by Wanke E. *et al*.^26^, spike trains and glial responses were simultaneously captured from individual sensing electrodes. Also using MEAs Fleischer *et al*.^27^ reported that astrocytes can be electrically stimulated and were shown to exhibited extracellular voltage fluctuations in a broad frequency spectrum (100–600 Hz). Ultra-weak signals^28^ and noisy electrical fluctuations generated by populations of glioma cells were also reported^29–31^. The strategy enabling the observation of these weak signals exploits large capacitive electrodes with a low intrinsic thermal noise.

In this contribution we demonstrate the potential of this ultra-sensitive electrical method, together with a micro-structured electrode surface, to measure extracellular ionic fluctuations in primary cultures of astrocytes. Comparatively to a typical MEA electrode, our sensing electrodes are few thousand times larger generating a thermal noise of 0.3 μV in the frequency window of 0.1 to 12.5 Hz. Although lacking spatial resolution, these electrodes provide unique conditions to capture the signals related to synchronization and cooperative dynamics produced by astrocytes.

In this paper we start by presenting the devices, the cells and the electrical methods used. Strategies to achieve an extracellular sensing electrode with a high sensitivity are discussed within the framework of an equivalent circuit of the cell/substrate interface. Then the intrinsic noise spectral characteristics of the electrodes are presented and discussed. The signal detection limit at a particular frequency is determined. The performance of flat bare gold electrodes is also compared with surfaces covered with a matrix of gold mushroom-like micro-structures. The additional increase in capacitance as well as the corresponding decrease in resistance introduced by the micro-patterned surface is quantified. Next, the use of micro-structured electrodes to record spontaneous activity generated by a primary culture of astrocytes is reported and the recorded signals are compared and validated with signals measured using optical fluorescence methods, reported by others. Finally, the relevance of the findings and their implications in the development of a tool to study slow and weak electrophysiological events are discussed.

## Results

### Description and characterization of the sensing electrodes

**Figure 1:**
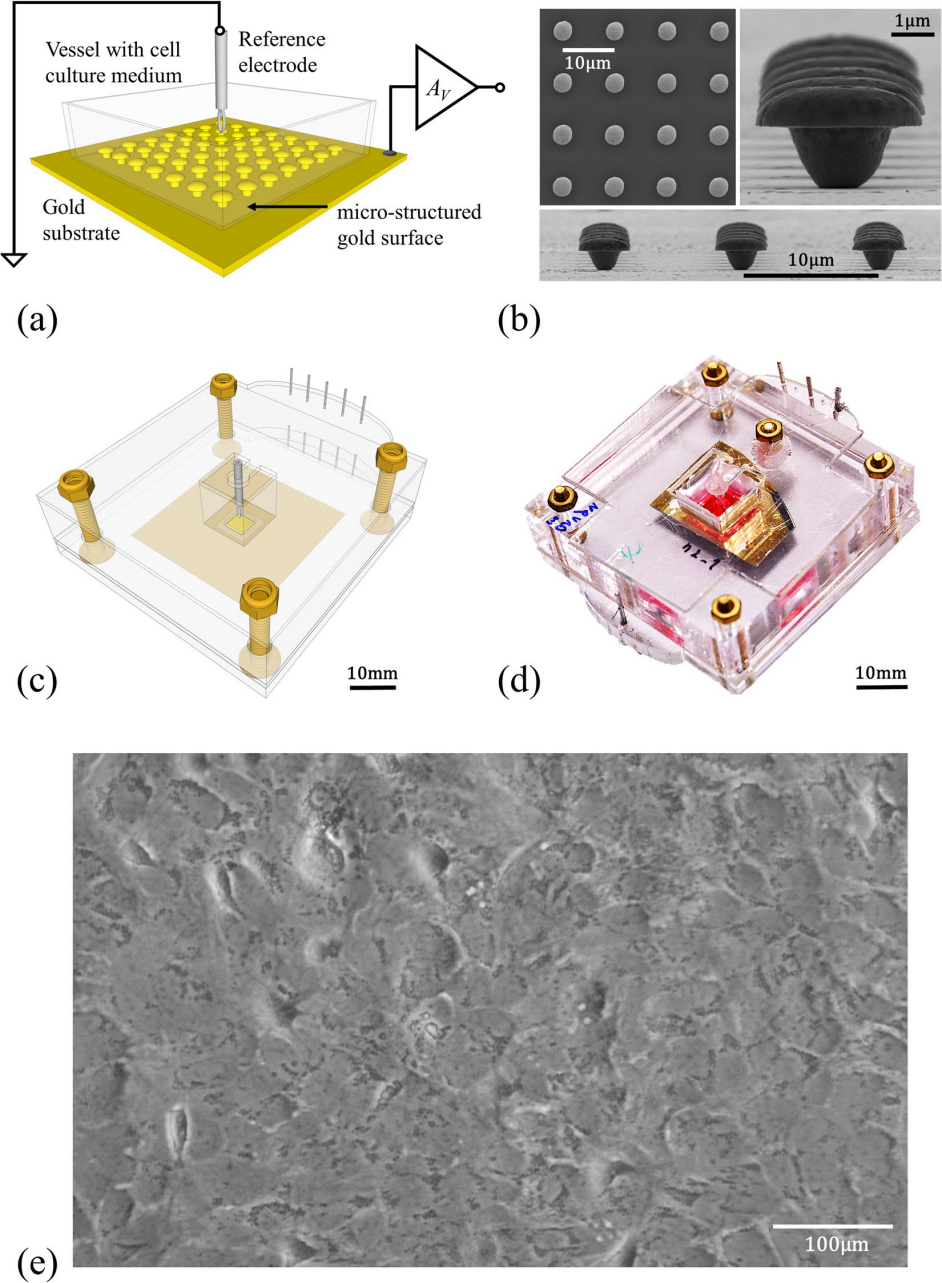
Sensing devices and cells used in this study. (**a**) Schematic diagram of the device, and electrical connections. (**b**) Scanning electron microscopy photographs of the gold mushroom-like structures. (**c**) Schematic view of the device holder. (**d**) Photograph of the complete sensing device. (**e**) Micrograph of a confluent population of astrocytes.

The basic structure of the sensing device together with the electrical connections and the cells is represented in Fig. 1. A schematic diagram of the gold mushroom-shaped electrode and the electrical connections to the voltage amplifier is represented in Fig. 1(a). Figure 1(b) shows a scanning electron microscopy photograph of the gold mushroom structured surface. The mushroom density is 4753 mushrooms per mm^2^. A schematic diagram of the integration of the sensing electrode in an acrylic vessel is represented in Fig. 1(c) and a photograph of a real device is shown in Fig. 1(d).

Measurements of the electrode impedance and electrical noise were first carried out to provide insight into the sensitivity of the electrodes and the advantages of using a micro-structured surface, in comparison with a flat gold electrode.

In order to understand how the information about the sensing electrodes is extracted we present and briefly discuss the equivalent circuit model used to represent the electrical coupling between the cells, the sensing electrode and the amplifier.

**Figure 2:**
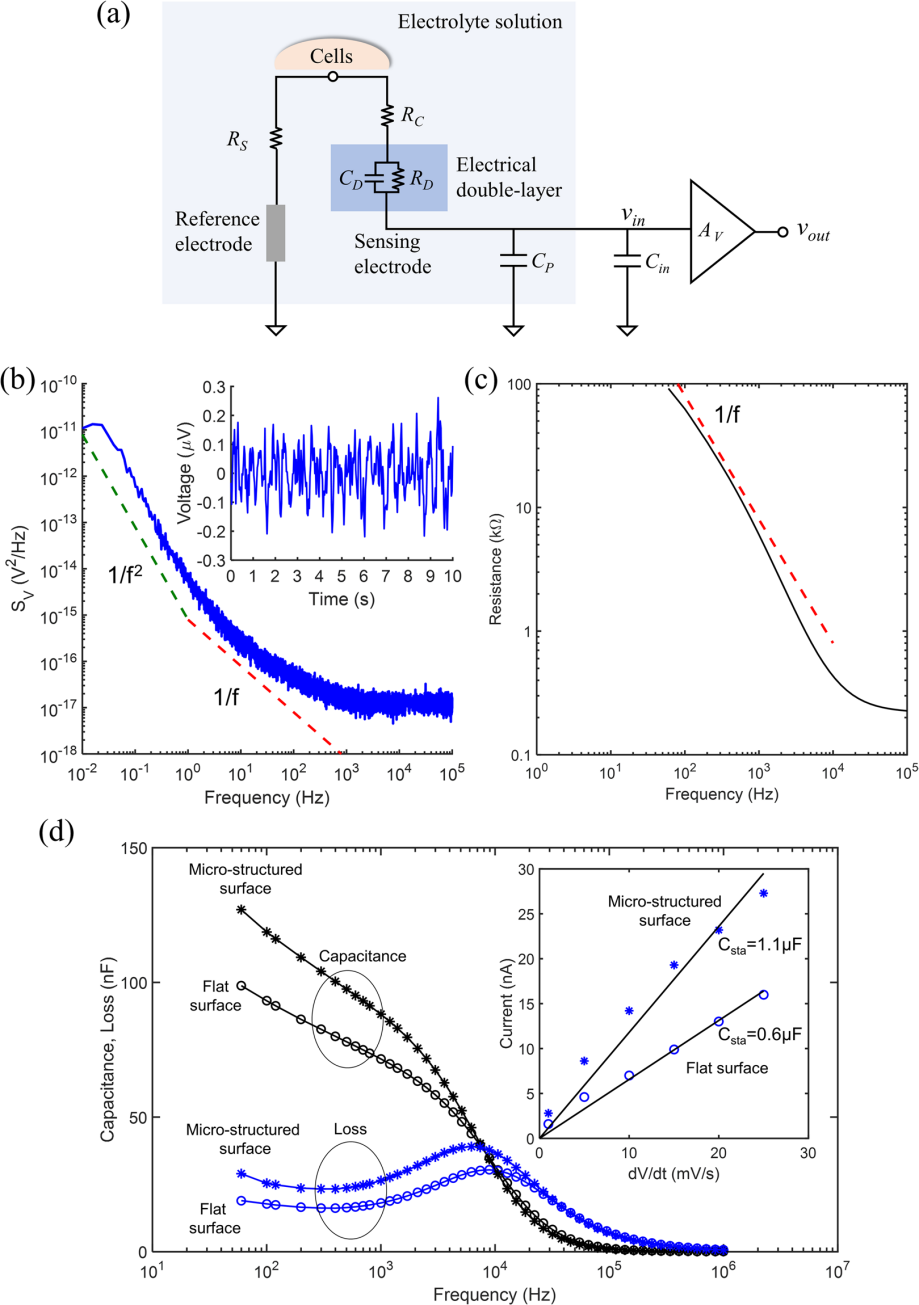
Electrical properties of the sensing electrodes. (**a**) Schematic diagram representing the electrical coupling between the cells and the measuring circuit. (**b**) Frequency dependence of the noise power density in voltage. (**c**) Frequency dependence of the total system resistance, *R*_*P*_. *S*_*V*_ was measured by dividing the full frequency range (10^5^ Hz) in several shorter ranges. The total smoothed power spectrum was obtained by joining the frequency segments. (**d**) Frequency dependence of the impedance components, Capacitance (*C*_*P*_) and Loss (*L*_*P*_ = 1/(ω*R*_*P*_)). The inset compares the displacement current measured for a micro-structured and for a flat electrode.

The equivalent circuit shown in Fig. 2(a) embodies the electrical coupling between the cells and the electrode. The electrode in contact with an electrolyte is modelled as the parallel between a capacitor (*C*_*D*_) and a resistor (*R*_*D*_). This is the electrical double-layer established at metal/electrolyte interface known as Helmholtz-Gouy-Chapman double-layer. *C*_*D*_ models the extent to which the electrode is polarizable and *R*_*D*_ takes into account the presence of Faradic currents. The parallel network of the double-layer appears in series with a spreading resistance (*R*_*C*_), which models the signal loss due to the cell-electrode distance. Usually, *R*_*C*_ is very small and can be neglected. The other circuit component are the seal resistance (*R*_*S*_), that models the cell adhesion to the electrode, the parasitic capacitance of the interface to the ground (*C*_*p*_) and the amplifier input capacitance (*C*_*in*_). This circuit is a simplified version of the standard model reported in literature^1,32,33^.

The equivalent circuit described above allows us to understand how the signal generated by the cell is coupled to the electrode. In particular, it shows which electrode physical parameters have to be optimized to achieve signal detection with maximum SNR ratio.

Assuming that the cells are adherent to the sensing electrodes, then *R*_*C*_ is very small and *R*_*S*_ is high. From a technological  point of view the parameters that we can optimize by adjusting the electrode design and materials are *C*_*D*_ and *R*_*D*_. The signal generated by the cells is an ac signal that couples to the sensing electrode trough *C*_*D*_. The higher is *C*_*D*_ the lower is the impedance and better is the electrical coupling between the  cell and the sensing electrode. In the absence of electrochemical reactions, *R*_*D*_ only contributes to the intrinsic thermal noise. The noise generated by the sensing electrode is usually higher than the noise generated by the amplifier. Therefore, *R*_*D*_ defines the noise floor and determines the signal detection limit.

The design rules for *R*_*D*_ and *C*_*D*_ are dependent on the type of amplification used. Signals can be amplified as current or as voltage signals. The case of current amplification, has been addressed in detail in a previous publication^34^. For current amplification the higher the value of *R*_*D*_ the lower is the thermal noise generated. In current amplification methods there is a trade-off between capacitance and resistance. The electrode area should be increased up to a point where the corresponding decreasing in resistance starts to add thermal noise and counterbalance the benefits of a high capacitance.

In this contribution we use voltage amplification. For this detection method, *C*_*D*_ should be maximized to lower the impedance and *R*_*D*_ should be low enough to generate a noise level below the noise generated by the voltage amplifier. The gold mushroom micro-structured surface allows us to maximize *C*_*D*_ and minimize *R*_*D*_ for a particular sensing area. As pointed out by other authors^19^ the microstructure also contributes to improve cell adhesion properties because the cells engulf the mushroom–like shapes and increase the seal resistance.

The power spectral density of the voltage noise, *S*_*V*_, as a function of frequency, is shown in Fig. 2(b). We use 10 times series to obtain the averaged noise. The dashed lines represent the power-law behaviour for reference. The magnitude of the noise decreases with the frequency, and for frequencies higher than 1 kHz the noise becomes frequency independent (white noise). For frequencies below 100 Hz, the noise follows a 1/*f* law that evolves for the lowest frequencies to a 1/*f* ^2^ frequency dependency (flicker noise). This frequency dependence is typical of electrodes in electrolyte systems^35–37^. The 1/*f* power law agrees with the frequency dependence of the system total resistance, *R*_*P*_, (see Fig. 2(c)), confirming that the intrinsic thermal noise of the resistive elements dominates the electrical noise of the recording system.

The inset in Fig. 2(b) shows a short time trace of the noise measured in the frequency band of 0.1 to 12.5 Hz. The voltage noise is 0.3 μV peak-to-peak and determines the detection limit of our measuring system for signals in the frequency range of a 0.1–12.5 Hz.

In Fig. 2(d) we present a comparison of the frequency response of the individual impedance parameters capacitance (*C*_*P*_) and resistance (*R*_*P*_) of a bare flat gold electrode with a mushroom covered surface. For convenience, the resistance (*R*_*P*_) is represented as Loss (*L*_*P*_ = 1/(ω*R*_*P*_)). Both *C*_*P*_ and *L*_*P*_ are strongly frequency dependent. This frequency dependence of the impedance has been extensively studied by us^31^ and others^38^ and was shown to be characterized by a Maxwell-Wagner dispersion, typical of electrodes immersed in electrolyte solutions. In Fig. 2 (d) this dispersion is located at 10 kHz and corresponds to the frequency position of the peak in the Loss curve. The dispersion occurs because electrodes immersed in electrolyte solutions give rise to electrochemical double-layers with high capacitances (*C*_*D*_) and high resistances (*R*_*D*_). This interface layer appears in series with the rest of the electrolyte solution. When compared with the double-layer, the bulk region has a lower capacitance (*C*_*B*_) as well as a  lower bulk resistance (*R*_*B*_). At low frequencies  the high capacitance layer dominates, however, and as the frequency increases the interfacial layer is progressively short-circuited and the low capacitive bulk layer starts to dominate the system response. The transition in frequency between the two layers is responsible for two capacitance plateaus shown in Fig. 2(d), one at low frequency and the other at high frequency. The values of *C*_*P*_ and *L*_*P*_ below the loss peak are related with the interfacial or double-layer region and the values above the relaxation peak are related with the bulk electrolyte solution. The relaxation frequency is mostly controlled by the bulk electrolyte resistance, *R*_*B*_^31^. Both flat and micro-structured gold electrodes were immersed in the same electrolyte solution, and therefore, as expected, the relaxation frequency must have the same value for both systems, as confirmed by the data in Fig. 2(d). The difference between the two electrodes is the surface topology; one surface is micro-structured and the other is flat. At 100 Hz, the micro-structured surface has a capacitance 30 nF higher than that of the flat surface. The corresponding double-layer resistance is also lower by 23 kΩ.

As shown in Fig. 2(d), neither  *C*_*P*_ nor  *L*_*P*_ reach steady state values at 100 Hz. Instead, they follow a trend to increasing values for lower frequencies. It is expected that the quasi-static capacitance (for *f* = 0 Hz) is significantly higher than the values measured at 100 Hz. The quasi-static values for *C*_*D*_ were obtained by measuring the displacement current *i*_*D*_ (*t*) in response to a slow voltage ramp *dv*/*dt*. The inset of Fig. 2(d) shows the displacement current for flat and micro-structured surfaces. The quasi-static capacitance (*C*_*sta*_) is estimated to be 0.6 μF for flat gold electrode and 1.1 μF for the micro-structured gold electrode. This represents an increase of 54% in the quasi-static capacitance. As discussed above, the higher the capacitance the higher the electrical coupling of the signal to the electrode. Previously, we have reported that, in voltage detection methods, the SNR increases linearly with the capacitance^34^. Therefore, assuming that the number of cells synchronized is constant, we would expect and increase in 54% in the SNR in comparison with a flat gold surface.

### Extracellular recording of Astrocytes signals

The performance of the gold mushroom-like electrodes was assessed using populations of astrocyte cells *in vitro*. The astrocytes were isolated from mouse cerebral cortex, as described in the materials and methods section.

**Figure 3:**
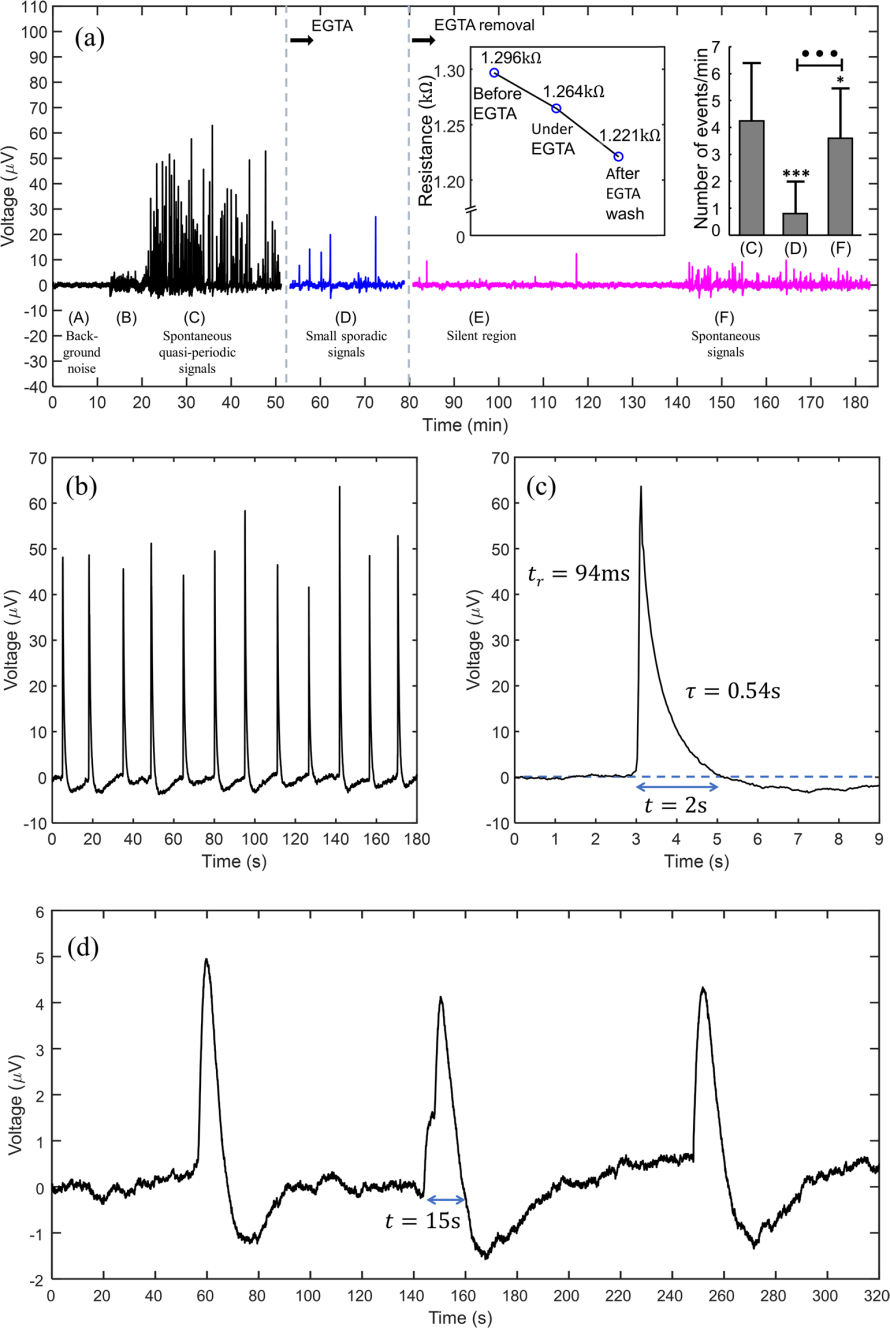
Electrical signals recorded in astrocytes populations. (**a**) An overview of a long-term recording of astrocyte population activity, with different experimental stages identified A-F. The inset shows how the high frequency (30 kHz) parallel resistance (*R*_*P*_) decreases upon the addition of EGTA. The histogram in the inset shows the number of signals per minute, before and after the addition of EGTA. (**b**) Time trace of a quasi-periodic activity recorded in the burst of activity labelled (A). (**c**) Detailed view of an individual signal in Fig. 3(b). (**d**) Long-lasting, low amplitude and sporadic signals. The experiments were conducted using a concentration of EGTA of 10 mM.

Figure 3(a) displays a typical time trace of the astrocyte electrical activity. Measurements were initiated after cell seeding. However, in the first hours only noisy fluctuations identical to bare electrodes (without cells) were measured. Cells only start to show a measureable activity approximately 3 hours after cell seeding. For simplicity, only a fraction of 20 minutes before the onset of activity is shown in Fig. 3(a). Cell covered electrodes show a 3–4 μV (peak-to-peak) noise fluctuation, as shown on region labelled (A). The onset of electrical activity is preceded by an increase on the average noise fluctuations (see the region labelled (B) on Fig. 3(a)). The electrical activity starts suddenly as a burst of discrete signals. The burst is comprised of quasi-periodic signals with a broad distribution in amplitudes that vary from 10 to 60 μV. However, for some relatively short periods, the signals can show a quasi-periodic behaviour with a frequency of approximately 0.1 Hz and average amplitude of 50 μV. Figure 3(b) shows an example of this quasi-periodic behaviour recorded inside the burst of activity labelled (C**)**. The burst of electrical activity was recorded for 30 minutes, after  which  the cell culture medium was replaced with an extracellular calcium-chelating agent (ethylene glycol-bis(β-aminoethyl ether)-N,N,N’,N’-tetraacetic acid; EGTA). The experiments were conducted using a concentration of EGTA of 10 mM. The histogram in the inset of Fig. 3(a) shows that in the presence of the calcium-chelating agent the frequency of signals lowers from an average value of 4 signals per minute (region (C)) to only 1 signal per minute (region (D)). Cells were kept in a medium with EGTA for 15 minutes, and then the electrolyte medium was replaced with fresh and normal cell culture medium. After the EGTA removal the astrocyte population remained silent, with occasional spikes. One hour after the EGTA removal, the astrocyte population re-started activity. This activity, labelled as region (F), is characterized by signals also spaced by an average time of 10 seconds, as observed on the initial burst of activity (region (C)), although the signal amplitude is lower. The reduction in amplitude may be due to residual EGTA on the cell culture surface. The experiment was repeated three times and in all cases the activity was substantially reduced upon addition of EGTA.

The addition of EGTA was conducted to inspect a possible relation between the observed signals and extracellular calcium waves commonly reported for astrocytes^39^. However, it has also been reported that the removal of calcium from the cell culture medium causes a disruption of the connections between cells (tight junctions) and also disturbs the cell attachment to the electrode surface^40–42^. In electrical terms, these perturbations on the tight junctions and cell adhesion properties will be reflected in a decrease in the seal resistance and imped the observation of signals. The tight junctions are re-established when the EGTA is washed and normal cell culture medium is used^40,42,43^.

In order to inspect for changes in the cell adhesion and inter-connecting properties, small-signal impedance measurements were carried out. The impedance of the cell/substrate interface is sensitive to changes in morphology, adhesion and inter-cell connecting properties. This approach is referred to as electric cell-substrate impedance sensing (ECIS)^44^. According to the ECIS model, the high frequency resistance should decrease when the tight junction is disrupted or when the cells are detached from the sensing electrodes^40^. The inset in Fig. 3(a) shows that the resistance measured at 30 kHz decreases from 1.296 kΩ to 1.221 kΩ, a decrease of 75Ω, which corresponds to a 5.7% decrease in resistance. Although this change in resistance is relatively small, it may be enough to impede the propagation of cooperative signals across the cells. Alternatively, if the disruption is affecting the cells attachment to the electrode, it may decrease the seal resistance and compromise the ability of our electrodes to record the signals.

Figure 3(b) shows a time trace of quasi-periodic signals inside the activity burst labelled (A) in Fig. 3(a). The signals are quasi-periodic, spaced by an average period of 10 sec. A detailed view of an individual signal is shown in Fig. 3(c). The signal shape is asymmetric and is characterized by a very fast time to peak (*t*_*r*_ < 94 ms) and a slower decay to base line (*t*_*d*_ = 0.54 s). The average width of the signal is *t*_*w*_ = 2 s. During the signal decaying to baseline, the current often overshoots to a lower value than the steady state background noise.

We also recorded ultra-slow astrocyte activity. Slow signals do not appear in bursts, but sporadically, in clusters of 3 or 4 signals. A typical example of a cluster of signals is shown in Fig. 3(d). Comparatively to the more rapidly varying signals, slow or long lasting signals are weaker, sparser in time (several minutes), and last longer (10 to 20 s). Long lasting signals only reach amplitudes with a peak value of 5 μV, 4 times smaller than a typical fast signal with average amplitudes reaching 20 μV.

In summary, astrocyte activity is characterized by discrete signals, with stereotyped shapes but different width, amplitude and frequency. High amplitude signals are narrow, appear in bursts and are quasi-periodic (f ≈ 0.1 Hz). Low amplitude signals are long lasting and appear sporadically. Digital video image of fluorescence from intracellular Ca^2+^ signals reported by other authors^45–47^, show signals that are alike, in shape, length and frequency, to the signals presented in Fig. 3. For example, a study by Kuga *et al*.^48^ report the observation of repetitive spikes with the periodicity of 1 minute, and lasting for approximately 10–20 seconds (similar to the ones in Fig. 3(d)). Other experiments^49^ using rat hippocampal astrocytes show spontaneous calcium transients with a frequency of approximately 1.3 Hz. Each individual Ca^2+^ signal lasts for approximately 10–20 s. A recent study^50^ using state-of-art image techniques in brain slices and *in vivo* showed astrocytes Ca^2+^ fluctuations lasting for approximately 14 s. The reported signals are also characterized by a fast rise time followed by a slower decay to the base line and a signal shape identical to the signals presented here.

## Discussion

The high-sensitivity of our measuring system is achieved by using large physical areas (4 mm^2^) further enhanced by an array of mushroom-like structures. In comparison with a flat surface, the micro-structured surface significantly lowers the interfacial impedance between the cell and the electrodes. The interfacial quasi-static capacitance is increased by approximately 54%. There is also a corresponding decrease in the interfacial resistance that brings the thermal noise to approximately 0.3 μV (peak-to-peak).

The effective area of a micro-structured surface is much higher than its total base level area, therefore, micro-structured surfaces require less physical occupation area when compared with flat surfaces that provide identical impedances. Larger area flat electrodes probe larger ensembles of cells, and this may not be desirable if only a fraction of the cells synchronize to generate signals, while the remaining cells contribute to noise and degrade the SNR. Therefore, the benefit of using micro-structured electrodes is the increase of the sensing electrode capacitance without the need to make very large base level area electrodes, hence, allowing a smaller population of cells to be probed. In addition to the obvious increase in capacitance, the group of M. Spira *et al*.^51^ had also shown that the cells engulf the mushroom shapes. This engulfment leads to an improvement of the electrical coupling between cells and the sensing substrate, modelled trough an increase in the seal resistance.

The electrode and the measuring methodology were evaluated by recording signals from astrocytes isolated from mouse cerebral cortex in primary culture. The measured signal is a superposition of the individual responses of several cells adherent on the electrode. We ascribe these quasi-periodic signals to the collective electrophysiological behaviour and synchronization of the activity across a population of cells on the electrode; thus, they arise from a cooperative phenomenon.

Although astrocytes do not fire action potentials, they are not electrically silent cells. Electrophysiological recordings using patch clamp methods revealed that astrocytic membranes are animated by very slow fluctuations that are intimately related to changes in neuronal activities^24,52–55^. Astrocytes do indeed exhibit a form of excitability, albeit one quite different from the known membrane electrical excitability of neurons. Astrocyte excitation is expressed as oscillations in cytosolic Ca^2+^ concentration and an increase in propagating waves of free Ca^2+^ increase^46,52,54,56^. This form of excitation is in line with the results reported herein where the astrocyte activity is partially inhibited by chelation of the extracellular Ca^2+^. Furthermore, the frequency and shape of the electrical extracellular signals match the ones using optical fluorescence probes reported in literature^55^. The oscillations occur at frequencies in the order of 0.1 Hz. Astrocytes excitability progress thousands of times more slowly than do their neuronal electrical counterparts. Activity waves spread from astrocyte to neighbouring astrocyte by means of gap junctions, which interconnect adjacent astrocytes into functional networks, both in brain tissues and under cell culture conditions^47,54,57^.

Although, the signals reported here have electrical characteristics in line with those reported as calcium waves, further experiments are required to establish an unambiguously link to calcium waves. The elimination of signals in the presence of calcium chelating agent EGTA is by itself not conclusive because calcium deprivation causes a disruption of the electrical coupling between cells and the sensing electrode as described in the Results section.

A search in the literature revealed that weak and slow signals, such as intercellular calcium waves, have been addressed using extracellular ion-selective probes. However, probes and optical methods have a low signal-to-noise ratio and may impact in the physiological functions of the cell membrane. Moreover, the lifetime of fluorescence dyes limits the observation to a few hours. Extracellular electrodes offer the advantage of being totally non-invasive and enable monitoring cells and tissues for periods of time as long as weeks.

The electrodes and the measuring methodology issued from our investigations and described herein are thus highly relevant for a community of neuroscientists and biologists interested in the study on how electrical signals are used by cells to communicate and coordinate their activities. There is a large body of evidence supporting the view that these slow signals are important regulators of cell behavior, controlling cell proliferation, migration, and differentiation^58,59^. Thus, while providing new tools, this study opens new directions for assessing biological events based on cooperative communication.

### Summary and conclusions

Our contribution presents a new approach for the detection and recording of ultra-weak and slow signals using extracellular microelectrodes. The ultra-sensitivity of our system was demonstrated by the recording of signals in primary cultures of cortical astrocytes populations. Understanding the physiological role and significance of this type of signalling requires more detailed investigations, but the electrodes and the methodology developed here represent already a powerful tool to reveal the dynamics of neuroglia ionic signalling. The integration of these real-time bioelectrical measurements with other methods will boost the understanding of how cells use bioelectrical signals to coordinate their activity.

## Methods

### (a) gold electrodes

To produce the gold mushroom-shaped microelectrode arrays, a thin layer of Cr (3 nm)/Au (40 nm) was first deposited on top of a silicon wafer (Si/SiO2) using magnetron sputtering. Then, Direct Write lithography (µPG 101 tool, Heidelberg Instruments; UV 400 nm, W = 10 mW @ 17%) was used to expose a mask constituted by 2.0 µm dots (in diameter) disposed in a square array (2 mm × 2 mm) with a lattice spacing of 10 µm. The samples were developed in a Microposit 351 solution for 60 s. Gold mushroom arrays were then obtained by electroplating inside the opened holes using a three-electrode configuration where Ag/AgCl was the reference electrode and a gold solution (Orosene E + 4gr/lt, for Italgalvano s.p.a.) under −1 V for 70 minutes at room temperature. This procedure led to a stalk height of 1.4 µm, a total mushroom height of 2.4 µm and a cap diameter of 3.8 µm. The stalk of the fabricated gold mushrooms is also seen to vary with the height, due to the profile induced on the photoresist during exposure.

### (b) Animals

C57Bl6/J mice were kept in our animal facility, with controlled temperature (21 ± 1 °C) and humidity (55%), with food and water *ad libitum* in a 12 h dark/light cycle. The experiments were performed in accordance with institutional and European guidelines (2010/63/EU) for the care and use of laboratory animals.

Both the Portuguese law (DL 113/2013) and the European law (directive 2010/63/EU) state that obtaining tissue for cell cultures without actually performing any procedures in a laboratory animal, as is the case in this paper, does not beg an official approval from the competent authority (Direcção Geral de Alimentação e Veterinária, DGAV), since no procedures are performed (the law understands that a procedure is the equivalent of provoking discomfort in an animal similar to a needle piercing the skin), only that the process of sacrificing animals is performed by a licensed user. Since the cultures are prepared from tissues extracted from animals euthanized by a licensed experimenter and the animals were kept in our licensed animal house facility, we stated that we performed the study according to the instituted guidelines both locally at our institute and by law.

### (c) primary astrocytes cultures

Primary mixed glial cultures were obtained from new-born C57Bl6/J mice with 0–3 days^60^. Briefly, after decapitation the brains were removed, and the meninges and cerebellum were discarded. Brain tissue was then mechanically dissociated and enzymatically digested (0.1% trypsin and 0.001% DNase I, 20 min at 37 °C). Cells were seeded in 25 cm^2^ or 75 cm^2^ flasks coated with poly-L-lysine, at a density of 0.2 × 10^6^ cells/cm^2^, and cultured in D-MEM/F12 with GlutaMAX^TM^-I supplemented with 10% fetal bovine serum, 0.25% gentamicin and 0.25 ng/ml M-CSF, at 37 °C and 95% air / 5% CO_2_ in a humidified incubator. Culture medium was replaced every 4 days and confluency was achieved after 15 days *in vitro*. Microglia and olidendrocyte precursor cells were removed by vigorous shaking resulting ed in astrocytes culture purity of approximately 98%.

After detachment of microglia cells, astrocytes were trypsinized (0.25%, 20 min at 37 °C) and seeded on the electronic devices.

An aliquot of 200.000 cells per cm^2^ was transferred to the well and was placed in an incubator (Thermo Scientific, Midi 40). Prior to cell deposition, the micro-structured electrodes were sterilized by UV treatment and the electrodes were coated with poly-L-lysine to promote cell adhesion. The cells were maintained at 37 °C in an incubator with a humidified atmosphere with 5% of CO_2_. The system assures the presence of enough cell culture medium to keep the cells viable over more than 24 hours without medium change. Cell numbers and viability were  assessed using a Neubauer chamber-based trypan blue live/dead exclusion assay.

Cells were incubated with D-MEM/F12 with GlutaMAX^TM^-I, supplemented with 10.0 mM ethylene glycol-bis(β-aminoethyl ether)-N,N,N’,N’-tetraacetic acid (EGTA). After 15 min, the EGTA medium was replaced with normal medium.

### (d) transducer holder

The transducer is based on 4 mm^2^ gold mushroom-shaped microelectrode arrays on a glass substrate. An acrylic vessel (see Fig. 1(c) and (d)) that could be filled with cells and cell culture medium was attached through an o-ring on top of the sensing gold substrate. A thin silver wire previously bleached to obtain an AgCl layer was used as reference electrode. The vessel was loosely covered with a lid to prevent evaporation of the medium.

After filling, the system was put into an incubator (Thermo Scientific, Midi 40). The total amount of medium supported by the holder is 1 mL, although it was used 400 µL, which assures the presence of enough cell culture medium to keep the cells viable over more than 24 hours without medium change.

### (e) electrical measurements

The entire experimental set-up was specifically designed for ultrasensitive detection. External interference was minimized through the use of a Faraday cage and low noise cables. Extracellular voltage measurements were carried out using a low-noise voltage amplifier (SR 560, Stanford Research) and a dynamic signal analyser (35670 A, Agilent). To minimize drift, the current amplifier is calibrated and the set-up is stabilized for at least two hours before measuring. The current was recorded as a function of time by using zero bias on the electrodes.

Small-signal impedance measurements were carried out using a RCL meter Fluke PM 6306. Quasi-static measurements were carried out using a picometer/voltage source (Keithley 6487).
